# Corrigendum: Scaling of Optogenetically Evoked Signaling in a Higher-Order Corticocortical Pathway in the Anesthetized Mouse

**DOI:** 10.3389/fnsys.2018.00050

**Published:** 2018-10-18

**Authors:** Xiaojian Li, Naoki Yamawaki, John M. Barrett, Konrad P. Körding, Gordon M. G. Shepherd

**Affiliations:** ^1^Department of Physiology, Feinberg School of Medicine, Northwestern University, Chicago, IL, United States; ^2^Department of Physical Medicine and Rehabilitation, Feinberg School of Medicine, Northwestern University, Chicago, IL, United States; ^3^Department of Bioengineering, School of Engineering and Applied Science, University of Pennsylvania, Philadelphia, PA, United States

**Keywords:** corticocortical, retrosplenial cortex, motor cortex, optogenetic stimulation, silicon probe

In the original article, there was a typographic error in Equation 2.

A correction has been made to the section *MATERIALS AND METHODS*, sub-section *Model Based Analysis*, paragraph 2, Equation 2:

(2) $$A_{M2}\left(t\right)=\vartheta \left(m\sum_{\Delta t=1}^{T}e^{\frac{-\Delta t}{\tau_{interact}}}A_{RSC}\left(t-\Delta t\right)-\theta \right)+c$$

The authors apologize for this error and state that this does not change the scientific conclusions of the article in any way.

The original article has been updated.

## Conflict of interest statement

The authors declare that the research was conducted in the absence of any commercial or financial relationships that could be construed as a potential conflict of interest.

